# UV-activated multilayer nanomatrix provides one-step tunable carbohydrate structural characterization in MALDI-MS†
†Electronic supplementary information (ESI) available: See DOI: 10.1039/c5sc00546a



**DOI:** 10.1039/c5sc00546a

**Published:** 2015-05-28

**Authors:** Rofeamor P. Obena, Mei-Chun Tseng, Indah Primadona, Jun Hsiao, I-Che Li, Rey Y. Capangpangan, Hsiu-Fong Lu, Wan-Sheung Li, Ito Chao, Chun-Cheng Lin, Yu-Ju Chen

**Affiliations:** a Institute of Chemistry , Academia Sinica , Taipei , Taiwan; b Department of Chemistry , National Tsing Hua University , Hsinchu , Taiwan . Email: yujuchen@gate.sinica.edu.tw; c Molecular Science and Technology Program , Taiwan International Graduate Program , Institute of Chemistry , Academia Sinica , Taiwan; d Department of Chemistry , National Taiwan University , Taipei , Taiwan; e Institute of Chemistry , University of the Philippines-Diliman , Quezon City , Philippines

## Abstract

The structure-specific fragmentation of gas-phase ions in tandem mass spectrometry among other techniques provides an efficient analytical method for confirming unknown analytes or for elucidating chemical structures. Using concentration-dependent UV-absorbing matrix-functionalized magnetic nanoparticles and matrix-assisted laser desorption-ionization mass spectrometry (MALDI MS), we developed a single-step pseudo-MS/MS approach for tunable ionization and fragmentation to facilitate structure determination. Without chemical derivatization, we have demonstrated that this approach successfully distinguished isomeric sets of di-, tri- and tetrasaccharides. Low concentration of nanomatrix provided an enhanced signal for accurate mass determination of the intact molecular ions of analytes present in the sample. In contrast, high concentration of nanomatrix induced extensive and unique fragmentation, including high-energy facile bond breakage (A- and X-type cross-ring cleavages), which facilitated the linkage and sequence characterization of oligosaccharides without conventional tandem mass spectrometric instrumentation. The practicality of this approach for complex sample analysis was evaluated by an oligosaccharide mixture, wherein molecular ions are unambiguously observed and signature product ions are distinguishable enough for molecular identification and isomer differentiation by this simple tunable approach. By probing the roles of the multilayer nanomatrix components: matrix (energy absorption), silane-coating (energy pooling and dissipation) and core Fe_3_O_4_ (fragmentation), a plausible energy transfer mechanism was proposed based on a computational study and photoelectron experiments. The differentiation of tri- and tetra-oligosaccharide shown in this study not only demonstrated the first step toward glycan characterization by nanoparticle-assisted MALDI-MS, but also shed some insight on the nanoparticle-mediated energy transfer dynamics behind our approach.

## Abstract 2

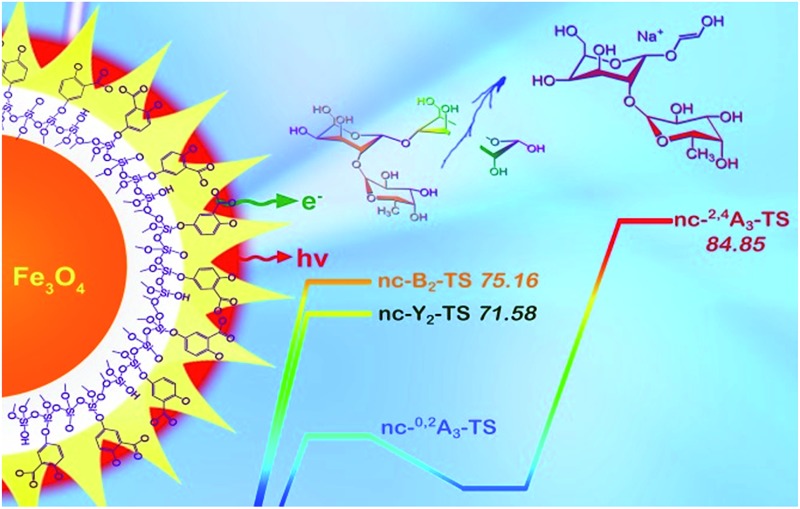
Our work highlights DHB@MNP-induced pseudo-MS/MS for oligosaccharide characterization, with some insights on this nanoparticle-mediated energy transfer dynamics.

## Introduction

Innovations in medicine and material science have inspired parallel developments in the synthesis of new chemicals and of various analytical methodologies. For example, the analysis of carbohydrates has gained considerable attention because of their close relationship with disease occurrence or progression, and because of their therapeutic potential.^1,2^ The functionality of carbohydrates depends on their high degree of isomeric diversity, which results from subtle changes in their unit assembly. Due to their intrinsic structural complexity, such as isomerism, linkage type, branching positions and branching types, the analysis of carbohydrates remains a formidable challenge.^3^ The rapid and unambiguous analysis of composition, sequence, linkage, branching and anomeric configuration and discrimination of isomeric saccharides are crucial for determining changes in carbohydrate architecture relative to their biological implications in diseased states.

Tandem mass spectrometry (MS/MS) is a valuable tool for determining empirical chemical formula and for characterizing molecular structures. Among the various biophysical methods, fragmentation by MS/MS or highly accurate mass by ultra-high resolution FTICR technique^4^ is the most efficient analytical platform for identifying unknown molecules or complex mixtures.^5,6^ Significant progress in sequencing to determine the structural differences between carbohydrates have been reported by various tandem MS/MS methods, including low-energy collision induced dissociation (CID) on a Q-TOF instrument,^7^ high-energy collision induced dissociation on MALDI TOF/TOF,^8^ and more sophisticated fragmentation techniques such as infrared multiphoton dissociation (IRMPD),^9^ electron capture dissociation (ECD)^10^ and sustained off-resonance irradiation (SORI) on Fourier-transform ion cyclotron resonance (FTICR) MS,^11^ and electron-transfer dissociation (ETD) on ion-trap MS.^12^ However, due to the low ionization of glycans, chemical derivatization (*e.g.*, permethylation or peracetylation) is generally required prior to characterization using MS/MS.^13^ Nevertheless, in most cases, the derivatization requires large amount of oligosaccharide (∼0.100 mg), which has limited its application for clinical application. In addition, derivatization may enhance the risk of introducing contaminants and side-reactions. The development of rapid, sensitive and comprehensive glycan sequencing methods that do not require chemical derivatization remains a considerable challenge. However, the development of such methods is imperative for establishing detailed structure–function relationships.

Nanomaterials have been reported to possess size-dependent properties, large surface area-to-volume ratios, high molar absorption coefficients and quantum properties that enhance efficient energy absorption and transfer to analytes.^14^ The interactions of lasers with different energy-absorbing nanoparticles, such as carbon nanotubes and Au metal nanospheres and nanoshells, have been explored in photodynamic therapy^15–17^ and laser medicine.^18,19^ In addition, these nanomaterial properties have been applied to nanostructure-assisted laser desorption-ionization mass spectrometry.^20^ It has been demonstrated that metallic nanoparticles can serve as matrix-assisted laser desorption-ionization mass spectrometry (MALDI MS) ionization matrices.^21–27^ Particularly, superparamagnetic iron oxide nanoparticles have distinct optical and electronic characteristics that influence their single and collective excitation responses to electromagnetic radiation.^28,29^ Recently, a glutathione-capped iron oxide nanoparticle has been reported to generate significant product ions for glycan sequence identification by MALDI MS in-source decay (ISD).^30^

**Fig. 1:**
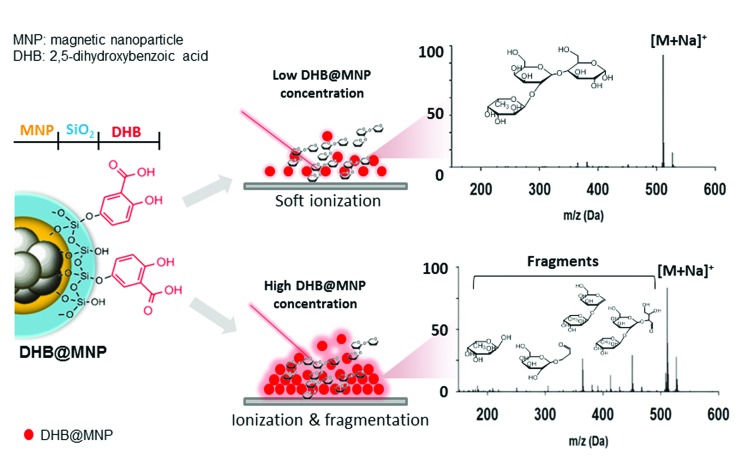
Schematic representation of tunable ionization and fragmentation by nanoparticle-assisted MALDI-TOF MS for structural determination of oligosaccharides; [M + Na]^+^ denotes sodium-adducted molecular ion peaks.

In this current work, we explored the unique energy absorption-transfer dynamics in multilayer (2,5-dihydroxybenzoic acid)-functionalized magnetic nanoparticles (DHB@MNP). The unique roles of each DHB@MNP layer in energy absorption and transfer synergistically provide a simple one-step, concentration-mediated, tunable ionization and fragmentation method for structural characterization of individual oligosaccharide as well as mixtures. At low concentrations, UV laser-excited DHB@MNP provides enhanced soft ionization for accurate mass measurements of intact molecular ions, whereas excess energy absorption and dissipation from high DHB@MNP concentrations causes harsh ionization, *i.e.*, the generation of an extensive but controlled degree of molecular fragmentation (Fig. 1). In addition to characterization of single analyte, notably, the DHB@MNP concentration-mediated process generated unique fragmentation fingerprints to successfully distinguish a series of isomeric trisaccharide and tetrasaccharide analytes as well as their mixtures. Moreover, the results of computational and photoelectron studies allowed us to propose a feasible mechanism underlying the nanoparticle-mediated energy-transfer dynamics from the multilayer components in the matrix@MNP. To the best of our knowledge, this work represents the first demonstration that nanomaterials facilitate successful molecular structure characterization for known oligosaccharide mixture analysis using a single MALDI-MS technique without conventional tandem (TOF/TOF) instrumentation, and has potential advantage as a complementary technique for elucidation of unknown compounds.

## Experimental

### Materials

Tetraethyl orthosilicate (TEOS, Sigma-Aldrich, Saint Louis, US), 2,5-dihydroxybenzoic acid (2,5-DHB, Waters) and 25% ammonia solution (Acros, Geel, Belgium) were used as received. Sucrose (Sigma-Aldrich), lactose (Sigma-Aldrich), 2′-fucosyl-d-lactose (Sigma-Aldrich), Lewis A trisaccharide (Le^A^, Calbiochem, Compton, UK), Lewis X trisaccharide (Le^X^, Merck, Darmstadt, Germany), Lewis Y tetrasaccharide (Le^Y^, Sigma-Aldrich) and Lewis B tetrasaccharide (Le^B^, Carbosynth) were purchased as oligosaccharide samples and used as received. Analytical grade methanol (Merck, Darmstadt, Germany) and 1-propanol (Acros) were used as solvents without redistillation. Deionized water (Direct-Q™, Millipore SA, Molsheim, France) was also used as the solvent for both the analyte and matrix.

### Synthesis and characterization of the matrix@MNP

The Fe_3_O_4_ magnetic nanoparticles (30 mg) were prepared by co-precipitation of Fe^2+^ and Fe^3+^ solutions in alkaline solution.^31^ In brief, FeCl_2_·4H_2_O (3.18 g) and FeCl_3_ (5.2 g) were dissolved in acid solution (25 mL ddH_2_O + 0.85 mL 12 M HCl), then it was added dropwise to an aqueous solution of 1.5 M NaOH (250 mL) to obtain a black precipitate. After washing with ddH_2_O twice, 0.01 M HCl (300 mL) was added, and the core MNP was redispersed (ddH_2_O). For the silanation process, the core MNP was treated with aqueous NH_3_ (25% w/w, 0.408 mL), ddH_2_O (0.3 mL) and TEOS (0.1 mL). The solution was stirred at 55 °C for 2 h until it yielded a black precipitate, SiO_2_@MNP. To synthesize the DHB@MNP, DHB (70 mg, 3 mL ddH_2_O) was added to the SiO_2_@MNP, followed by addition of 0.01 M HCl (1.5 mL) at room temperature for 12 h. After centrifugation (8000 rpm, 15 min) and washing (ddH_2_O), the DHB@MNP were dried and stored at 4 °C for characterization and future use.

Transmission electron microscopy (TEM) images (see ESI 7, Fig. S4†) of the MNPs were obtained by a Philips Tecnai F20 G2 FEI-TEM electron microscope (FEI Co., Hillsboro, OR, USA) with 0.14 nm lattice resolution, 200 kV accelerating voltage, and ×50 to 1.5 × 10^6^ magnification. The sequential surface modification of the MNPs was unambiguously confirmed by FT-IR spectra (Perkin-Elmer, Waltham, MA, USA). The crystal structure of the nanoparticles was determined by XRD analysis using a multifunction high-power XRD Bruker R8 Discover SSS (Bruker AXS LTD, Coventry, UK).

### Sample deposition

The DHB@MNP (2 mg) nanoparticles were suspended in 0.1 mL of ddH_2_O to afford a 20 000 μg mL^–1^ stock solution and further diluted to obtain 100, 200, 300, 400, 500, 1000, 5000 and 10 000 μg mL^–1^ solutions for subsequent MALDI-TOF MS analysis. The oligosaccharides (0.5 mg) were dissolved in 0.5 mL of ddH_2_O to afford 1 mg mL^–1^ solutions. After mixing 0.5 μL (100 fmol) of the oligosaccharide solutions with 0.5 μL of the DHB@MNP solutions, the resulting mixture was spotted onto a stainless steel sample plate, completely air dried and analyzed using MALDI-TOF MS. The Fe_3_O_4_ MNP, SiO_2_@MNP and commercial 2,5-DHB solutions were prepared and used as matrices by following the above procedures.

### Mass spectrometry analysis

MS analyses were performed using MALDI-TOF MS (4800, Applied Biosystems, Foster City, CA) equipped with an Nd-YAG laser (355 nm) operating at a repetition rate of 200 Hz. The spectra were recorded in reflectron mode using an accelerating voltage of 20 kV and a grid voltage of 16 kV in the MS/MS mode using CID gas (air). For accurate mass measurements, the instrument was calibrated with known standards (2,5-DHB and Angiotensin 1 for MS and MS/MS, respectively) to obtain an accuracy of 20 ppm. The MS/MS measurements were conducted using a collision energy of 1 kV and a CID gas pressure of 3.7 × 10^–6^ torr. A typical mass spectrum was obtained by averaging 500 laser shots, and the data were processed and analyzed with the Data Explorer software (Applied Biosystems, Foster City, CA). For the assignment of each product ion peak of all the oligosaccharides, we employed three criteria: (a) mass accuracy, ≤10 ppm, (b) signal-to-noise ratio, *S*/*N* ≥ 10, and (c) resolution, *R* ≥ 2000 to resolve the isotopic pattern.

### Computational detail

The conformational space of 2′-fucosyl-d-lactose with a sodium cation was explored using Replica Exchange Molecular Dynamics (REMD) method implemented in AMBER 9.^32^ Three different Na^+^ binding modes to the trisaccharide were used as starting structures for the conformational search; (1) Na^+^ binding to fucose and glucose, (2) Na^+^ binding to fucose and galactose, and (3) Na^+^ binding to galactose and glucose. A set of temperatures was generated using a temperature predictor for parallel tempering simulations^33^ (300.0, 368.1, 448.5 and 543.5 K) to give an exchange probability of 0.3. Prior to REMD, four replicas of the trisaccharide were each equilibrated to its target temperature for 2 ns with a time step of 0.002 ps using Langevin dynamics with *γ* = 5 ps^–1^. All bonds involving hydrogen atom were constrained using the SHAKE algorithm. Non-bonded interactions were calculated using a cutoff distance of 12 Å. The Glycam06 parameters were used with 1–4 scaling of the electrostatic and van der Waals interactions designated to 1.^34^ For the actual REMD simulations, 20 ns was used and structures were sampled at every 2 ps. Swaps between replicas of neighboring target temperature were attempted every 1 ps. All sampled trajectories at 300 K were extracted from the REMD simulations and were minimized using MM method. Structures within 6 kcal mol^–1^ of the global minimum of a given trajectory were then fully optimized using the DFT method. All geometries were fully optimized without any constraints, and energetics of the stationary points on the potential energy surface were calculated at the M06-2X/6-31+G(d) level. Frequency calculations were performed on all structures to confirm that the reactants, intermediates, and products had no imaginary frequencies and that transition states possessed only one imaginary frequency. Relative energies were corrected for vibrational zero-point energies (ZPE, not scaled). All of the DFT calculations were performed with the GAUSSIAN 09 package of programs.^35^

## Results and discussion

### Nanomatrix concentration dependence of ion/fragment generation

**Fig. 2:**
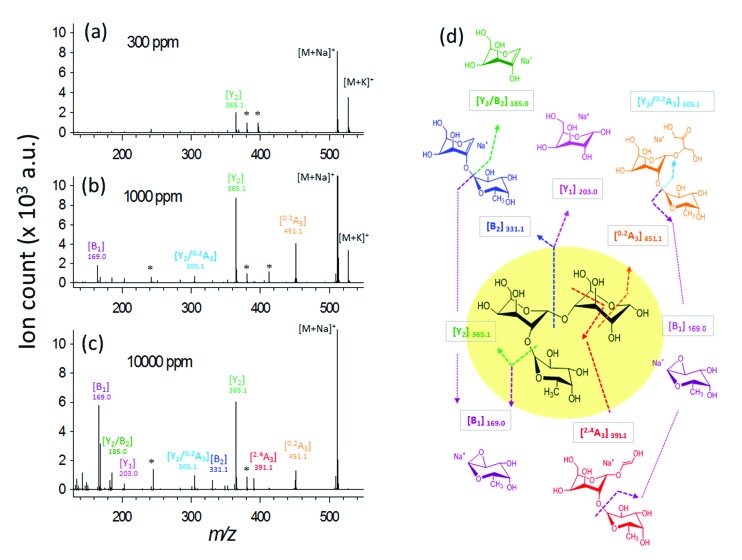
Ionization behavior of 2′-fucosyl-d-lactose. (a–c) Full spectra at 300, 1000 and 10 000 μg mL^–1^ DHB@MNP. The DHB-derived (“*”) peaks are shown. (d) Fragmentation pattern of 2FDL. Product ion assignment is based on Domon and Costello nomenclature.^36^

To demonstrate the simultaneous preservation of intact molecular ions and generation of fragment ions in the nanoparticle-assisted MALDI process, the Lewis trisaccharide 2′-fucosyl-d-lactose (2FDL), which consists of three monosaccharide units (fucose, galactose and glucose), was selected as a model compound. At a low DHB@MNP concentration (300 μg mL^–1^), the 2FDL sodium- and potassium-adducted molecular ion peaks, [M + Na]^+^ (*m*/*z* = 511.1) and [M + K]^+^ (*m*/*z* = 527.1), were predominantly present with relatively clean background (Fig. 2a). Product ions which gave full structural information on 2FDL were observed and described below. For detailed product ion assignment, the Domon and Costello nomenclature^36^ was used (ESI 1†).

**Fig. 3:**
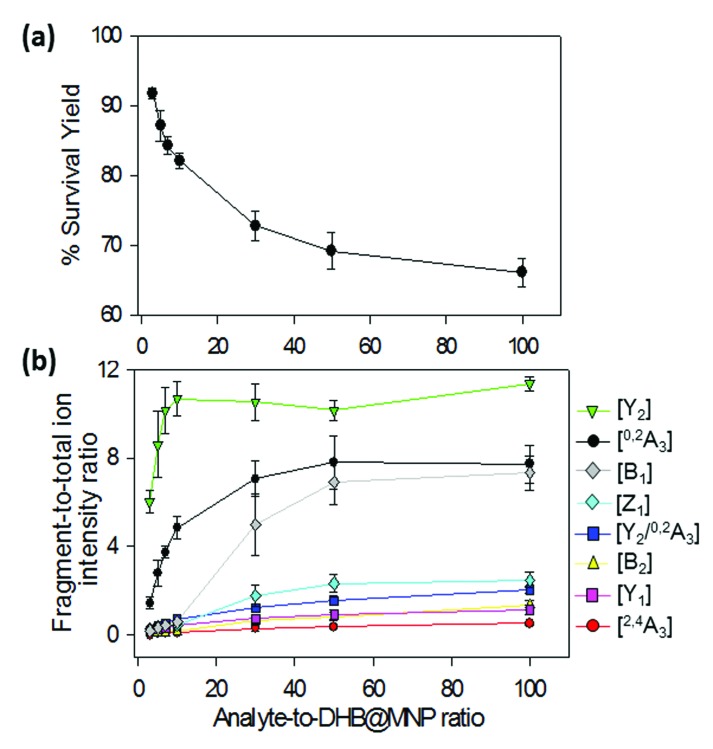
(a) Survival yield and (b) percent (%) product ion-to-total ion ratio for 2FDL. The steep decline in parent ion survival yield between 100–1000 μg mL^–1^ (1–10 analyte-to-DHB@MNP ratio) (a) is accompanied by sharp rise in intensities of fragment ions (b), particularly ^0,2^A_3_ and Y_2_. Survival yield (a) is defined as the sum of abundances of the precursor ions, [M + Na]^+^ and [M + K]^+^, divided by the sum of the abundances of all analyte-derived ions, including the precursor ions and all fragments.

When the DHB@MNP concentration was increased (from 300 to 1000 μg mL^–1^), extensive fragmentation was observed in the three product ions: ^0,2^A_3_ (*m*/*z* 451.1, cross-ring dissociation at C0–C2 position), Y_2_ (*m*/*z* 365.1) and B_1_ (*m*/*z* 169.0) (Fig. 2b). Additional fragments [derived from glycosidic bond dissociation (B_2_, *m*/*z* 331.1; Y_1_, *m*/*z* 203.0; Y_2_/B_2_, *m*/*z* 185.0)] and cross-ring fragment ions [Y_2_/^0,2^A_3_ (*m*/*z* 305.1)] began to appear at 1000 μg mL^–1^. These fragment ions could not be produced using either the free DHB matrix alone or a high power laser (ESI 2, Fig. S1†). Moreover, these product ions yielded relatively good signal-to-noise ratio (*S*/*N* = 260–5395) and well resolved peaks (*R* = 4790–7227). In order to evaluate the extent of DHB@MNP-dependent fragmentation, survival yield, which gives a quantitative view of either the precursor ion dissociation or the fragment ions generation, was calculated to give the relative abundance of each oligosaccharide-derived ion at DHB@MNP different concentration. The survival yield of precursor ion was calculated according to the equation:
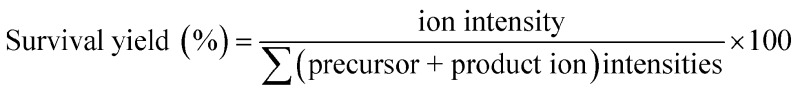


The fragmentation degree was assessed by dividing the intensity of each fragment ion with the sum of the intensities of all oligosaccharide-derived ions. Both were plotted as a function of analyte-to-DHB@MNP ratio (Fig. 3a and b, respectively).

We observed that both precursor ion dissociation and fragment ion generation is proportionally dependent on DHB@MNP concentration. As shown in Fig. 3a, the 2FDL parent ion begins to sharply decline starting at 90% between 100–1000 μg mL^–1^ (1–10 analyte-to-DHB@MNP ratio) nanomatrix and gradually levels at 65% between 3000–10 000 μg mL^–1^ (30–100 analyte-to-DHB@MNP ratio). As the DHB@MNP concentration was increased (100, 300, 500, 1000, 3000, 5000 and 10 000 μg mL^–1^), the fragmentation degree for the various product ions increases as shown by gradually enhanced fragment-to-precursor ion ratio trend for the various fragments (Fig. 3b). When the DHB@MNP concentration was further increased to 10 000 μg mL^–1^, additional ^2,4^A_3_ (*m*/*z* 391.1), B_2_ (*m*/*z* 331.1) and B_1_ (*m*/*z* 169.0) fragments were generated. This semi-quantitative correlation demonstrates that “soft” ionization (preservation of the intact molecular ion) and “harsh” ionization (generation of product ions) could be simultaneously achieved in simple MS mode by varying the concentration of the DHB@MNP nanomatrix.

To better understand this phenomenon, we compared the mass spectra of the 2FDL product ions generated using the one-step DHB@MNP-assisted MALDI MS method with those generated using conventional tandem MS/MS methods, including the MALDI-PSD, MALDI-CID, ESI Ion-Trap and ESI Q-TOF (ESI 3, Table S1†). In all of these different fragmentation methods, fragments resulting from glycosidic bond cleavage due to fucose (Y_2_) and glucose (B_2_) loss, cross-ring cleavage of the glucose moiety (^0,2^A_3_), or glycosidic and cross-ring cleavages (Y_2_/^0,2^A_3_) are commonly observed (Fig. 2b). The B_2_ and Y_1_ fragments from the cleavage of the glycosidic bond in galactose are easily generated in both of the MALDI MS/MS (PSD and CID) ionization methods. Interestingly, cross-ring cleavage fragments ^2,4^A_3_ are only formed through DHB@MNP-assisted MALDI MS (ESI 3, Table S1† and Fig. 2b). In addition, a less common fragmentation pathway (Fig. 2b) from further Y_2_ glycosidic bond cleavage producing galactose (Y_2_/B_2_) and fucose (B_1_) fragments was uniquely observed at high DHB@MNP concentration (>10 000 μg mL^–1^) (Fig. 2c) and was absent in the other conventional MS/MS methods.

### Theoretical basis of fragmentation energetics

The ^2,4^A_3_ fragment was only observed when using DHB@MNP and not when using conventional MS/MS approaches. In addition, products from the cleavage of the glycosidic bond (B_1_, Y_2_) were produced in great abundance relative to the B_2_ and Y_1_ products (Fig. 3b). This result implies that the two dissociation pathways may require different activation energies. To determine the energetics of the aforementioned fragmentation pathways, we conducted theoretical calculations.

The optimized lowest-energy conformer found for 2FDL in the cyclic hemiacetal form (c-2FDL [Na^+^]) at the M06-2X/6-31+G(d) level is shown in Fig. S2a.† Here, the sodium ion is held by four oxygen atoms within a distance of 2.4 Å. Furthermore, the cross-ring cleavage of oligosaccharides are reported to require the non-cyclic aldehyde form (Fig. S2b†) at the reducing-end of the sugar rather than the cyclic hemiacetal form.^37^ Therefore, we also calculated the 2FDL non-cyclic form nc-2FDL[Na^+^] (Fig. S2b†).

**Fig. 4:**
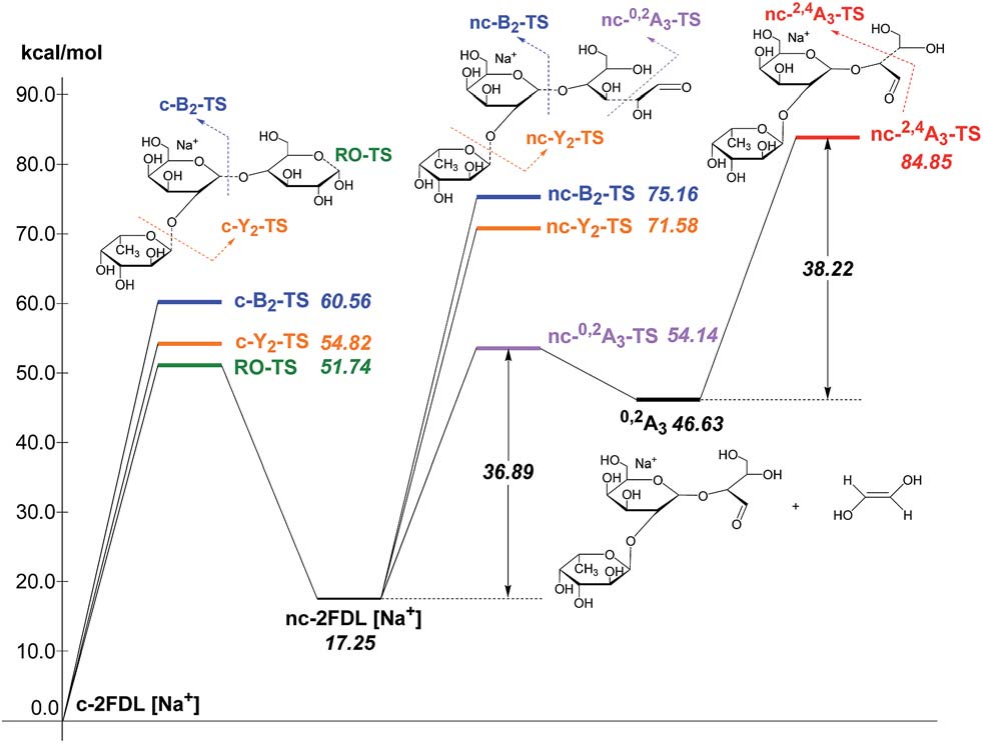
Energy profile of fragmentation at the M06-2X/6-31+G(d) theory level with zero-point correction; RO-TS = ring opening transition state; RO-product = ring opening product.

The calculated transition state energies of the fucose/galactose glycosidic bond cleavages and cross-ring cleavages were plotted against the cyclic hemiacetal form c-2FDL [Na^+^] (Fig. 4). We found that the transition state of the fucose–glycosidic bond cleavage (cyclic Y_2_-transition state, c-Y_2_-TS; 54.82 kcal mol^–1^) has a lower energy than that of the galactose cleavage (cyclic B_2_-transition state, c-B_2_-TS; 60.56 kcal mol^–1^). For the non-cyclic 2FDL transition states, a similar trend was observed for the fucose and galactose cleavages: the nc-Y_2_-TS energy (non-cyclic Y_2_-transition state, 71.58 kcal mol^–1^) is lower than the nc-B_2_-TS energy (non-cyclic B_2_-transition state, 75.16 kcal mol^–1^). These computational results are consistent with our experimental results (Fig. 2a), in which the fucose cleavage pathway for generating Y_2_ and B_1_ ions is more readily observed than the galactose cleavage pathway for generating B_2_ and Y_1_ ions, presumably due to the lower activation barrier imposed by the acidic hydrogen in fucose (Scheme S1a and S1b, ESI 5†). The overall activation barrier for the fragmentation pathway producing ^0,2^A_3_ is comparable to that of Y_2_. To produce ^0,2^A_3_, c-2FDL is first converted to the non-cyclic form through a ring-opening transition state (RO-TS, 51.74 kcal mol^–1^), which has a lower energy than the cyclic-Y_2_ transition state (c-Y_2_-TS). The transition state for the subsequent cross-ring cleavage to form ^0,2^A_3_ (nc-^0,2^A_3_-TS, 54.14 kcal mol^–1^ relative to c-2FDL, Fig. 4) is similar in energy to c-Y_2_-TS (54.82 kcal mol^–1^). With the six-membered-ring transition states for fragments ^0,2^A_*n*_ and ^2,4^A_*n*_,^32^ the activation barriers for the individual cross-ring cleavage steps are 36.89 and 38.22 kcal mol^–1^ for the generation of ^0,2^A_3_ and ^2,4^A_3_, respectively (Fig. 4; see Schemes S1c and d† for more details regarding the fragmentation pathways). The corresponding transition state (nc-^2,4^A_3_-TS; 84.85 kcal mol^–1^) for the uniquely observed ^2,4^A_3_ is located at the highest energy level in the overall energy profile (Fig. 4). Overall, the theoretical results indicated that the generation of the B_2_, Y_1_, and particularly the ^2,4^A_3_ fragments requires a higher energy barrier than that required for the generation of the Y_2_, B_1_ and ^0,2^A_3_ fragments. This finding supports the hypothesis that DHB@MNP-induced dissociation could provide sufficient energy for inducing extensive fragmentation of 2FDL in the gas phase.

### Role of multilayer nanomatrix in energy transfer

In an attempt to gain insights into the mechanism behind the energy supply and transfer process by the nanomatrix (DHB@MNP), we examined the multilayer components of magnetic nanoparticles (DHB@MNP, SiO_2_@MNP, core MNP) for their ability to simultaneously ionize and induce fragmentation of 2FDL at 300 and 10 000 μg mL^–1^ nanoparticle concentrations, respectively.

The use of naked core MNP at 300 ppm, *i.e.*, without either silane or DHB coating, promoted significant dissociation of the precursor (Fig. S3a†). Interestingly, the cross-ring products, including ^0,2^A_3_ and particularly the unique product ^2,4^A_3_ produced with much higher energy barrier, were more abundant than the energetically favorable Y_2_ fragment. However, the core MNP also introduced background ions in the mass spectrum. The additional silane coating on MNP (SiO_2_@MNP, Fig. S3b†) significantly reduced such background, yet also caused approximately 40% lower ionization efficiency of 2FDL. With further conjugation of ionization agent, DHB matrix, on SiO_2_@MNP (abbreviated as DHB@MNP), the precursor ion intensity was restored (Fig. S3c†). As expected, increasing the DHB@MNP concentration (10 000 μg mL^–1^) produces many fragments, including the most abundant fragments from fucose glycosidic bond cleavage (Y_2_, B_1_), cross-ring cleavage such as the ^0,2^A_3_, ^2,4^A_3_ and Y_2_/^0,2^A_3_, as well as minor fragments from galactose glycosidic bond breakage (Y_1_ and B_2_) (Fig. S3d†). Based on the comparison of fragment intensities observed by core MNP, SiO_2_ layer and DHB coating, it is clear that core MNP produced significantly more fragments, especially those with high energy barrier, which further suggested that core MNP plays major role for energy absorption and transfer, resulting in thermal energy increase and dissipation into the analyte, inducing spontaneous dissociation. Overall, the different nanoparticle components (DHB, silane-coating and core MNP) contribute synergistically to the absorption, generation and transfer of energy to the analyte. As shown by our observation above, the silane shell prevents the fragmentation of MNP and functions as a “thermal sink”.^38^ On the other hand, the presence of matrix (DHB) molecules augments the ability of the nanoparticles to mediate the energy transfer process and the spontaneous dissociation at high concentration. These properties afford simultaneous ionization and product ion generation, which is not feasible with the free matrix alone (Fig. S1a–f†).

### Photoelectron-assisted fragmentation by UV-activated DHB@MNP

**Fig. 5:**
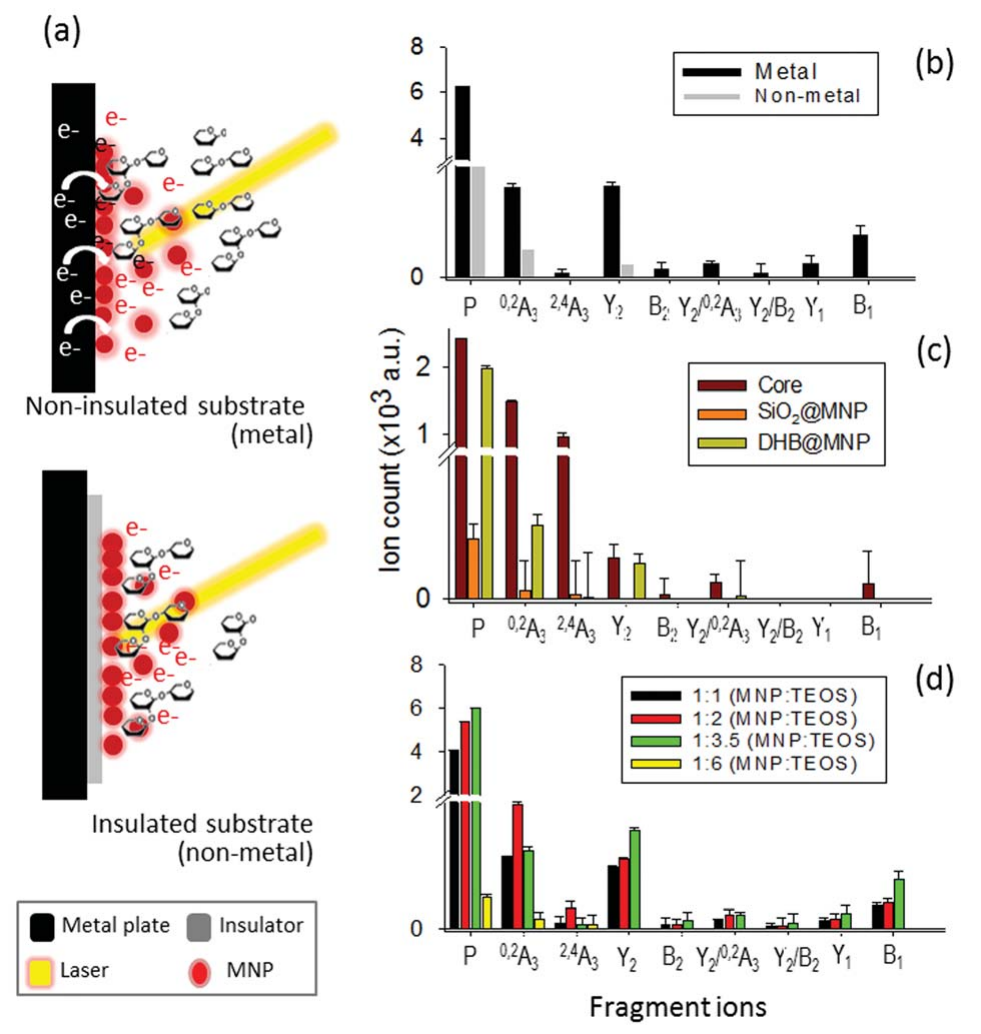
(a) Photoelectron-assisted fragmentation by DHB@MNP. (b) Influence of the substrate (metal *vs.* non-metal). (c) Source of photoelectron in the MNP and role of each nanoparticle component. (d) Effect of thickness of silane-coating; P denotes precursor ions.

In light of the empirical and theoretical results presented so far, we hypothesize that besides the generation of high thermal power by the nanomatrix to improve ionization and to induce fragmentation, the excited photoelectrons may play a key role in the fragmentation of the analyte ion.^39,40^ To verify our hypothesis, we investigated the source of photoelectrons, including the potential emission of photoelectrons from either the DHB@MNP or MALDI sample wells (stainless steel), and their correlation with the observed MNP-induced fragmentation process. We first evaluated the effect of photoelectrons from the DHB@MNP to induce the fragmentation process. To exclude additional sources of photoelectrons, we insulated the MALDI sample wells with electric tape (0.18 mm thickness) before spotting the sample and DHB@MNP, and then compared the results to the uninsulated metal target (Fig. 5a).

**Fig. 6:**
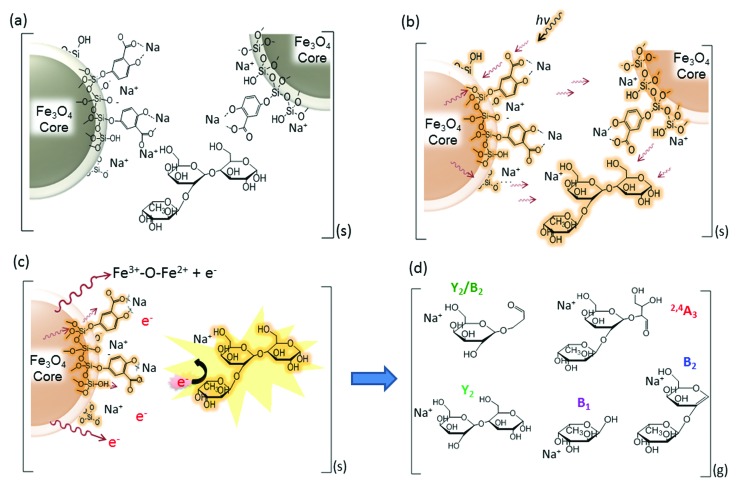
Proposed energy transfer mechanisms of the nanoparticle-assisted MALDI for the ionization and fragmentation of oligosaccharides. (a) Alkali-metal adduct formation in DHB@MNP in the condensed phase; the neutral oligosaccharide is sandwiched between the crystallized nanoparticles. (b) Absorption, conversion (to heat) and pooling of energy by the DHB@MNP and heat transfer to the oligosaccharide molecule. (c) Desorption of the oligosaccharide into the plume and highly energetic collision with ejected electrons from MNP. (d) Fragmentation of oligosaccharide into Na^+^-adducted fragment ions.

As shown in Fig. 5b, the total ion count of both precursor and fragment ions were reduced by three- and five-order, respectively, after insulation. The results suggested that the photoelectrons from the MALDI plate and DHB@MNP synergistically contributed to the ionization and fragmentation of 2FDL analyte. In insulated condition, as shown in Fig. 6c, the core MNP generated dramatically more and intense fragments than SiO_2_@MNP (>1000-fold) or DHB@MNP (6-fold). It is noteworthy that the unique ^2,4^A_3_ fragment ion was only produced by core MNP and greatly diminished by either SiO_2_@MNP or DHB@MNP (Fig. 5c). Thus, the results revealed that among the multi-layer components of the DHB@MNP, the photoelectrons from core MNP play the major role to induce dissociation, and the metal substrate contributes to this electron pool in the MALDI process.^41^ In the solid state, the surface of iron oxide undergoes intervalence charge transfer (IVCT)^42^ and rapid electron delocalization (electron hopping)^43,44^ leading to orbital vacancies and migrating electron–hole pairs known as excitons,^44^ which may also help account for the observed photoelectron-induced dissociation in this study.

Based on the observed >1000-fold reduction of fragment ions by SiO_2_@MNP (Fig. S3b† and 5c), we surmise that the silane network might have “shielded” the MNP-derived photoelectrons from escaping towards the surface of the nanoparticle. To test our hypothesis, the silane thickness was varied by changing the MNP : TEOS ratio (1 : 1, 1 : 2, 1 : 3.5 and 1 : 6) of DHB@MNP (Fig. S4†) and its effect was examined upon the model molecule on insulated MALDI substrate. As expected, the “thinner” coating (<0.2 nm thickness, Fig. S4a–c†) generated a higher number of fragments and higher peak intensities (Fig. 5d). On the other hand, the thickest silane coating (1 : 6, MNP : TEOS, ≈5 nm thickness, Fig. S4d†) generated the lowest peak intensity of precursor ions and least degree of fragmentation; product ions have >400-fold decrease on summed peak intensity (Fig. 5d), suggesting a “shielding” effect by the silane coating. Moreover, the observed trend also suggests that the silane shell may serve as a thickness-dependent “silane shield” from molecular fragmentation due to the presence of photoelectrons induced by MNP.

### Mechanism of energy transfer in nanomatrix-assisted ionization and fragmentation in MALDI MS

Based on the characteristic multifunctional role of DHB@MNP and the theoretical results on the analyte dissociation energetics, we propose a model for the energy absorption and transfer mechanisms to account for the ionization and dissociation in nanomatrix-assisted MALDI MS.

The first step begins with the formation of a complex between the carboxylate group on the DHB@MNP and Na^+^ in the condensed phase (step a, Fig. 6a). The sodium-adducted ion has been reported to be the major channel for the formation of the carbohydrate precursor ion,^45^ and our study also confirmed that the removal of Na^+^ by 15-crown-5 ether before MALDI deposition reduced the majority of the precursor ion (ESI 8†). After mixing and drying the sample (oligosaccharide) and nanomatrix on the plate, the sodium-adducted oligosaccharide is potentially trapped through coordination with either the salicylate on the silane shell or the carboxylate ligand on DHB within the crystallized nanoparticles. Upon laser irradiation (step b, Fig. 6b), the nanomatrix absorbs, converts and pools the energy, causing the entire nanoparticle to “heat up”. The “sandwiched” oligosaccharide molecule absorbs this heat and subsequently desorbs to the gaseous plume (step c, Fig. 6c). Furthermore, the mobile photoelectrons produced from the MNP core, which provides the Fe^3+^–O–Fe^2+^ redox couple (Fig. 6c), are activated and desorbed. In the multiphoton ionization model, a matrix molecule transforms into a radical cation *via* the absorption of ≥2 laser photons and subsequently emits electrons.^46^ In our case, we observed that the photoelectrons emitted from the MNP and MALDI plate played a major role in inducing unique analyte fragmentation (Fig. 5). In addition, the absorption of the laser radiation by the iron oxide surface has been reported to cause excitation and the spontaneous emission of electrons, *i.e.*, photoexcitation and photoemission.^47^ As these “hot” photoelectrons collide with the oligosaccharide, the precursor ions spontaneously dissociate into their fragment ions (Fig. 6d) in a charge-remote process,^45^ resulting in the formation of alkali metal-adducted product ions and intact precursor ions in the gas phase.

The amount of ions generated during the ionization process is proportional to the amount of energy absorbed and transferred to the analyte.^48^ Hence, depending on the amount of energy absorbed, the molecule may either form a stable molecular ion or undergo spontaneous dissociation. At high DHB@MNP concentrations, considerably more photoelectrons and DHB molecules are present. Thus, increasing the nanomatrix concentration provides a parallel increase in the density of highly energized electrons in the plume, which may in turn supply the extra internal energy required to cause spontaneous dissociation of the oligosaccharide molecules upon collision.

### Differentiating isomeric oligosaccharide mixtures by DHB@MNP-induced fingerprint patterns

Finally, we evaluated the practicability of our approach for complex sample analysis at two levels. Firstly, we carefully examined the common and fingerprint fragments for the differentiation of three sets of isomeric trisaccharides and tetrasaccharides. Secondly, to extend the application of this approach for mixture analysis, accurate mass determination and DHB@MNP-induced fingerprint pattern were employed to identify the individual components in an oligosaccharide mixture containing both an isomer pair and two non-isomeric compounds with different molecular masses.

**Fig. 7:**
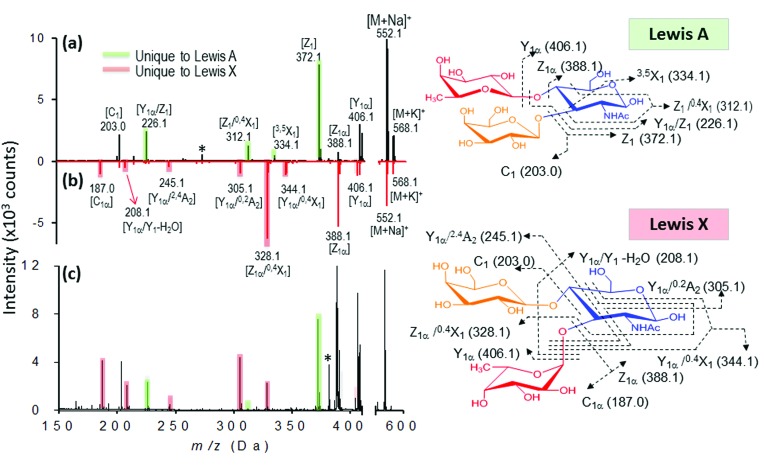
Identification of individual isomeric oligosaccharides (a and b) and mixture (c). (a) MALDI mass spectra of Lewis A (a, black) and Lewis X (b, red). The fingerprint ions unique to Lewis A and Lewis X are highlighted in green and red, respectively. (c) Mixture analysis of Lewis A and Lewis X based on their fingerprint ions.

As shown in our results, several glycosidic and cross-ring dissociation fragments that were characteristic of each isomeric structure were observed (Table S2†). In the first set of trisaccharide pair, 2FDL and 3FDL (3-fucosyl-d-lactose), the unique cross-ring product ions Y_1α_ – H_2_O (*m*/*z* 347) and ^0,2^A_3_ – C_2_H_5_O (*m*/*z* 405) served to distinguish 3FDL (ESI Fig. S6, see summary in Table S2†). The second isomeric trisaccharide pair, Lewis A and X, both contain an *N*-acetylglucosamine (GlcNAc) residue at their reducing ends but differ in their linkage position. As shown in Fig. 7, Lewis X is linked at C-3 by α-l-fucopyranose (Fuc) and at C-4 by β-d-galactopyranose (Gal), whereas Lewis A is substituted at C-3 and C-4 by Gal and Fuc, respectively. These diagnostic ions include the characteristic Z fragments for Lewis A, *m*/*z* 372.1 (C-3-linked Gal dissociation), and the characteristic Y_1α_/^0,2^A_2_ fragment for Lewis X, *m*/*z* 305.1 (combined loss of O-3-linked Fuc and ^0,2^A_2_ cross-ring cleavage), as shown in Fig. 7a and b. However either 1–3 or 1–4 linkage loss generates isobaric Z and Y fragment ions, which are non-structurally unique markers, and the previously reported semi-empirical approach by MALDI-PSD^49^ and electrospray ion trap mass spectrometry^50^ may fail to differentiate the isomeric Lewis-type oligosaccharides. As shown in Fig. 7a and b, the nanomatrix generated extensive structurally unique cross-ring fragmentation, *i.e.*, A- and X-type fragments, for both isomers. For Lewis A, further dissociation of Z_1_ [*m*/*z* = 372.1], to generate Z_1_/^0,4^X_1_ [*m*/*z* = 312.1, additional GlcNAc cross-ring cleavage], and Y_1α_/Z_1_ [*m*/*z* = 226.1, O-4-linked Fuc loss] indicated the sequence and glycosidic linkages. Under the same conditions, Lewis X underwent even more extensive dissociation from the relatively stable cross-ring cleavage to structurally identify it, including [Z_1α_/^0,4^X_1_, *m*/*z* = 328.1], [Y_1α_/^0,2^A_2_, *m*/*z* = 305.1], [Y_1α_/^2,4^A_2_, *m*/*z* = 245.1], [Y_1α_/Y_1_ – H_2_O, *m*/*z* = 208.1] and [C_1α_, *m*/*z* = 187.0]. For the third isomeric tetrasaccharide pair, the unique cross-ring-type fragmentations were also clearly observed to differentiate the Lewis B and Lewis Y (Table S2, see also Fig. S7 and S8†). As shown in Fig. S7,† ^0,4^X_1_-type cross-ring dissociation occurred to generate the fingerprint fragments of Lewis B, including Z_1α_/^0,4^X_1_ [*m*/*z* = 474.1, O-4-linked Fuc loss and GlcNAc cross-ring cleavage] and Y_1_/^0,4^X_1_ [*m*/*z* = 328.1, combined Fuc/Gal loss and GlcNAc cross-ring cleavage]. For Lewis Y (Fig. S8†), fragment Z_1_ [*m*/*z* = 372.1] unequivocally revealed that Fuc–Gal is O-4-linked to GlcNAc, a linkage that could be further confirmed by O-3-linked Fuc loss to yield Z_1α_/Y_1_ [*m*/*z* = 226.0]. By the use of the fingerprint fragment ions (Table S2†), each of these oligosaccharides was distinguished from their counterparts in the neat mixture, which were mostly cross-ring fragments.

Interestingly, we observed that A-type fragments only occurred as a signature pattern to determine Gal1-4 GlcNAc linkage, which may be due to the dissociation-susceptible Fuc1-4GlcNAc linkage.^51^ Glycosidic bond cleavages are charge-induced, but cross-ring cleavages are charge-remote processes, *i.e.*, energy dependent.^52^ In MALDI PSD, glycosidic bond cleavages (Y and B) are slow reactions (kinetically driven), whereas cross-ring cleavages are rapid reactions (thermally driven).^53^ Although such fragmentation reactions could also be observed using other ionization methods, the combination of both glycosidic and cross-ring cleavages could only be observed using the nanomatrix approach. Thus, we concluded that the energy activation barrier could be overcome easily by the synergistic energy transfer mechanism that was unique to the multifunctional nanomatrix.

We further evaluated the broader applicability of our approach for a trisaccharide mixture based on the facile generation of isomer-characteristic fragment ions by the nanomatrix-mediated MALDI MS. In this case, we chose the isomeric trisaccharide pair Lewis A and X as our model. As shown in Fig. 7c, when both Lewis A and Lewis X are present in the mixture, the Lewis A fragment fingerprint pattern, including the three characteristic Z series fragments, Z_1_, Z_1_/^0,4^X_1_ and Y_1α_/Z_1_. (Fig. 7c, green shadowed peaks) are unambiguously observed. Similarly, five characteristic fragments of fingerprint ion of Lewis X, including Z_1α_/^0,4^X_1_, Y_1α_/^0,2^A_2_, Y_1α_/^2,4^A_2_, Y_1α_/Y_1_ – H_2_O and C_1α_ can distinguish it from Lewis A. The fragment patterns unique to either Lewis A or Lewis X unambiguously identified the presence of both isomers in the mixture.

**Fig. 8:**
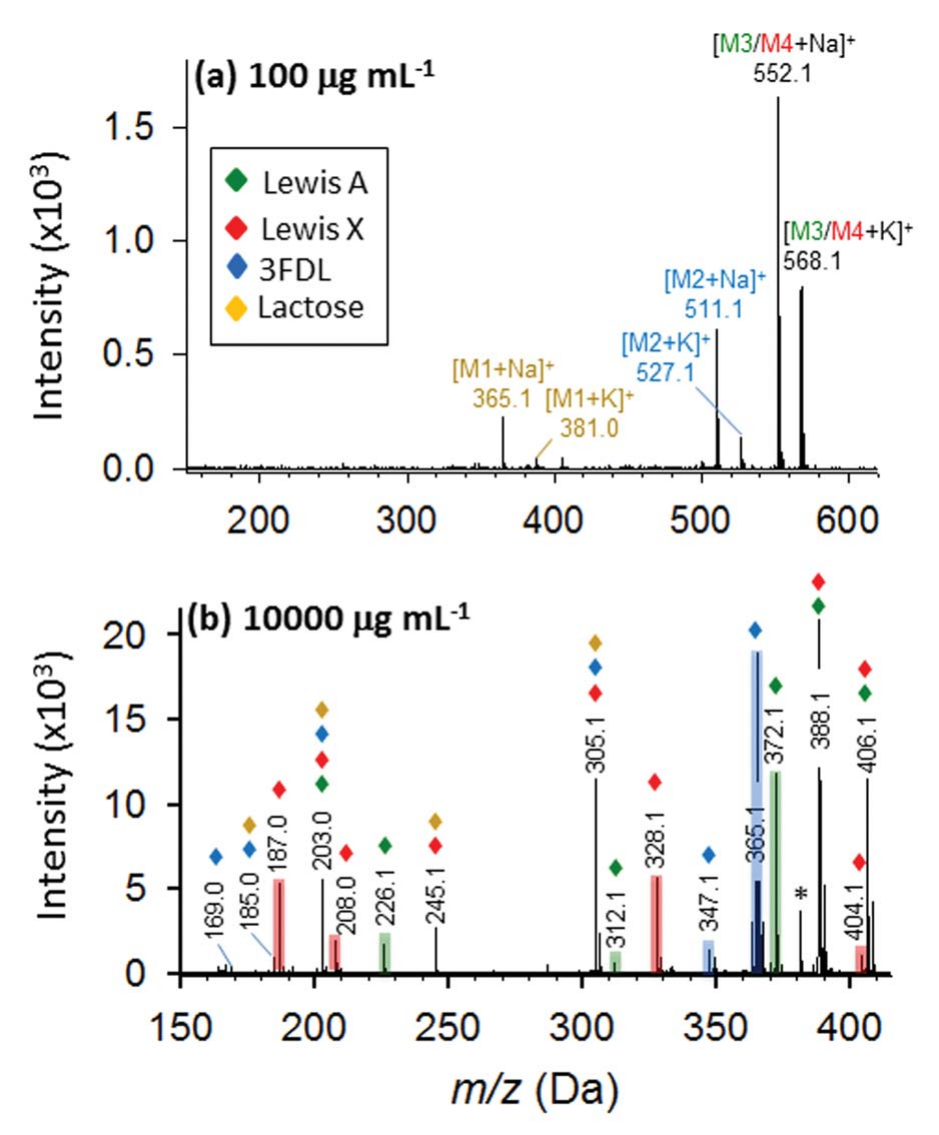
Identification of oligosaccharide mixture containing lactose (brown), 3-fucosyl-d-lactose (blue), and isomeric Lewis A (green) and Lewis X (red). Full MALDI MS spectra at 100 (a) and 10 000 μg mL^–1^ (b) DHB@MNP. The fingerprint ions unique to 3-fucosyl-d-lactose, Lewis A and Lewis X are highlighted in blue, green and red, respectively.

Furthermore, we evaluated a more complex case for mixture of four oligosaccharides including lactose, 3-fucosyl-d-lactose (3-FDL), and two isomers, Lewis A and Lewis X. In synthetic chemistry, characterization of a particular compound includes measurement of its accurate molecular mass ≤5 ppm, which is sufficient to confirm the molecular formula. At low concentration (100 μg mL^–1^) of the nanomatrix, three types of saccharides are definitely present by the sodium- and potassium-adducted precursor ion shown in Fig. 8a. By accurate mass measurement within 5 ppm accuracy, we are able to match the potential presence of lactose (theoretical mass: 365.1060, 3.3 ppm) and 3-FDL (theoretical mass: 511.1638, 1.8 ppm), respectively. Similarly, the peak present at *m*/*z* 552.1921 suggests the potential presence of either sodium adducted Lewis A or Lewis X (theoretical mass 552.1904) or their mixture.

However, at low nanomatrix concentration, the isobaric mass could not provide sufficient information to distinguish the isomeric saccharides, Lewis A and X, from each other. This information can be found at high nanomatrix concentration (10 000 μg mL^–1^, as shown in Fig. 8b), wherein the signature fragment ions verified the identity and differentiation of the isomeric Lewis A (Z_1_, Z_1_/^0,4^X_1_ and Y_1α_/Z_1_) and Lewis X (Z_1α_/^0,4^X_1_ and Y_1α_/Y_1_ – H_2_O) (for detailed fragment ions refer to Table S3†). Although, some isobaric peaks (*e.g. m*/*z* 203, 305) are also present in the spectrum (Fig. 8b), this only indicates some similarity in chemical composition of lactose, 3-FDL, Lewis A and Lewis X. Taken together, the results for the unambiguous identification of the isomeric Lewis A/Lewis X, as well as the four oligosaccharide mixture, demonstrated the feasibility of analyzing complex samples by the one-step tunable fragment fingerprinting in MALDI MS without conventional sample pre-derivatization step (*e.g.*, permethylation and peracetylation)^46^ or tandem mass spectrometry.^44,45^

## Conclusion

In the current work, we have presented a simple one-step ionization and fragmentation platform for structural characterization and fingerprint-based analysis of a series of isomeric oligosaccharides and mixtures. A low concentration of UV laser-excited DHB@MNP provided an enhanced signal for accurate molecular weight determination. By tuning high DHB@MNP concentrations, extensive analyte dissociation occurs, including glycosidic bond breakage and cross-ring cleavage to determine unambiguously the glycan sequence and linkage. Moreover, by the use of theoretical calculations and photoelectron experiments, we explored the synergistic actions of the nanomatrix’s multilayer components (Fe_3_O_4_ core MNP, SiO_2_ and DHB) as energy absorbers, converters and poolers, simultaneously ionizing and inducing structure-specific analyte fragmentation.

The applicability of this one-step method was demonstrated successfully by distinguishing linkage isomers, including isomeric tri- and tetrasaccharides, based on their characteristic and comprehensive fragmentation patterns. The feasibility for complex sample analysis was demonstrated on a proof-of-concept isomer mixture model. Our approach successfully distinguished the sequence and linkage of glycan stereoisomers, which represents a major challenge in current glycomic and glycoproteomic analyses. We expect that these unique fragmentation patterns can be used as a simple approach for rapid and high-throughput characterization of synthetic carbohydrates. With further exploration towards preservation of fragile carbohydrate molecular ions by soft ionization and full carbohydrate sequencing for more challenging structural features, such as polyLacNAc extension and branching, sulfation and sialylation linkages, we intend to broaden our understanding on the finer structural details of more complex and biologically relevant carbohydrates.

## Author contributions

Y.J.C., R.P.O and M.C.T. conceived the project, designed the experiments, discussed the results and implications, and commented on the manuscript at all stages. Y.J.C., R.P.O., M.C.T., I.C. and C.C.L. co-wrote the paper. R.P.O. and M.C.T. contributed equally, and I.P. and I.C.L. performed the MALDI-MS experiments. I.P. and R.Y.C. synthesized and characterized the nanoparticles. J.H., H.F.L. and W.S.L. performed the theoretical calculations. All authors commented on the manuscript. All authors have given approval to the final version of the manuscript.

## Conflict of interest

The authors declare no competing financial interests.
