# Supplementary material for: UV-activated multilayer nanomatrix provides one-step tunable carbohydrate structural characterization in MALDI-MS
Source: Chem Sci. 2015 May 28;6(8):4790–800. doi: 10.1039/c5sc00546a (PMC5502396; doi:10.1039/c5sc00546a)
Supplement: Supplementary file 1 [file SC-006-C5SC00546A-s001.pdf]

Electronic Supplementary Material (ESI) for Chemical Science.
This journal is © The Royal Society of Chemistry 2015

Supporting Information

UV-activated multi-layer nanomatrix provides one-step tunable carbohydrate structural characterization in MALDI-MS

Rofeamor P. Obena,‡[ae] Mei-Chun Tseng,‡[a] Indah Primadona,[bc] Jun Hsiao,[a] I-Che Li,[d] Rey Y. Capangpangan,[bc] Hsiu-Fong Lu,[a] Wan-Sheung Li,[a] Ito Chao,[a] Chun-Cheng Lin,[b] Yu-Ju Chen*[bd]

1. Domon and Costello nomenclature 2
2. Influence of matrix amount and laser intensity on ionization and fragmentation (**Figure S1**) 3
3. Comparison of different ionization methods with DHB@MNP-assisted MALDI-MS (**Table S1**) 4
4. Optimized structures of 2-FDL (**Figure S2**) 5
5. Fragmentation pathways for 2-fucosyl-D-lactose. (**Scheme S1**) 6
6. The mass spectra obtained by multilayer functional nanoparticles. (**Figure S3**) 7
7. Effect of silane thickness on the DHB@MNPs **(Figure S4)** 8
8. Alkali metal ion-dependent stability of precursor and fragment ions. (**Figure S5**) 9
9. Differentiation of isomeric oligosaccharides by DHB@MNP-assisted MALDI MS (**Table S2, Table S3, Figure S6, Figure S7, Figure S8, Figure S9**) 10
10. References 14

1. Domon and Costello nomenclature

Domon and Costello introduced the nomenclature pertaining to fragmentation of carbohydrates.[S1] According to this nomenclature, the ions retaining the charge at the reducing terminus are designated as X for cross-ring cleavages, and Y and Z for glycosidic bond cleavages. Those retaining a charge at the nonreducing terminus are designated as A for cross-ring cleavages, and B and C for glycosidic bond cleavages. Sugar rings are numbered from the nonreducing end for A, B, and C ions and from the reducing end for the others. Greek letters are used to distinguish fragments from branched-chain glycans, with the letter α representing the largest branch. In the case of ring cleavages, superscript numbers are given to show the ruptured bonds. In addition, ions produced as a result of more than one cleavage are designated with a slash between sites of cleavages.

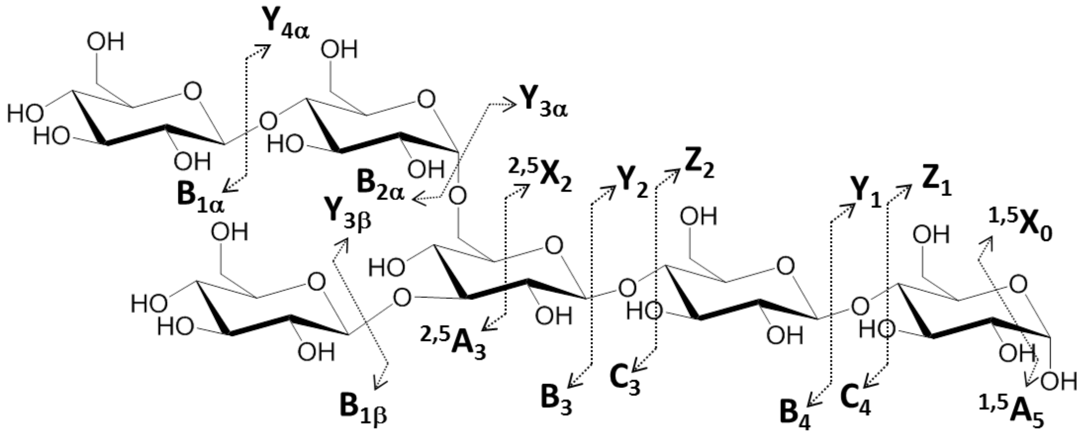

2. Influence of matrix amount and laser intensity on ionization and fragmentation

By the conventional MALDI MS method using DHB, the molecular ions are overwhelmed by background peaks especially at high matrix concentration (Figure S1a-f). It is also noteworthy that no fragment ions from cross-ring cleavage were observed in the spectra by this method. On the other hand, low laser power (Figure S1g-h) was not enough to generate molecular ion peaks; whereas at higher laser power, sodium- and potassium-adducted peaks together with matrix-derived peaks began to appear (Figure S1i-l). Likewise, no analyte-derived cross-ring fragment ions were observed in these spectra. Note: The glycosidic bond cleavage peak intensity is relatively low compared to the matrix-derived peaks for identification.

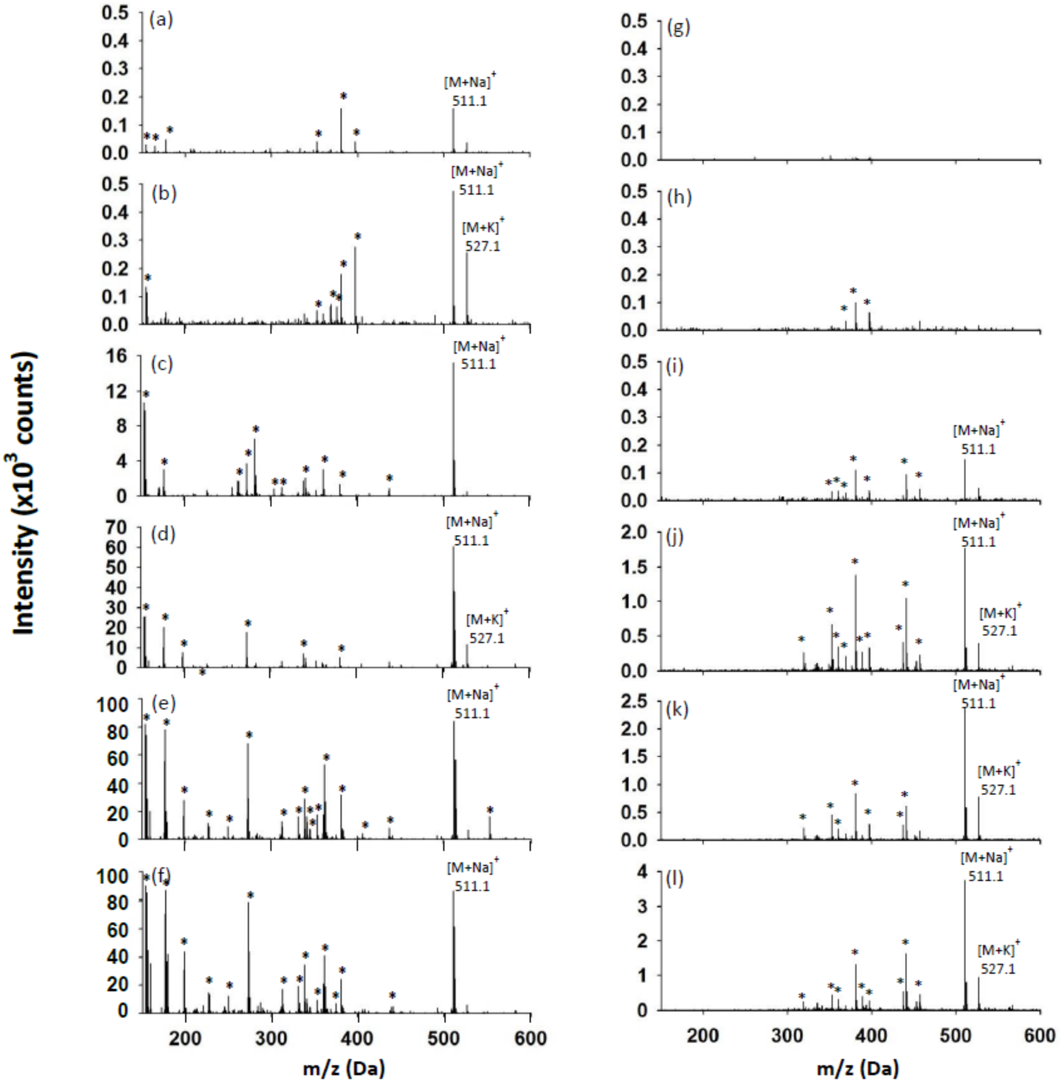

Figure S1. Influence of matrix amount (a-f) and laser intensity (g-l) on ionization and fragmentation. DHB amount: (a) 300, (b) 500, (c) 1000, (d) 5000, (e) 10000, and (f) 20000 μg mL^{-1} DHB. Laser intensity: (g) 4800, (h) 5200, (i) 5600, (j) 6000, (k) 6400, and (l) 6800 a.u. [M+Na]$^+$ and [M+K]$^+$ are sodium and potassium adducts. (*) denotes DHB matrix-derived peaks.

3. Comparison of different ionization methods with DHB@MNP-assisted MALDI-MS

Table S1. Product ions of 2'-fucosyl-D-lactose generated by DHB@MNP-assisted MALDI MS versus various MALDI and ESI MS/MS methods.

Method	Parent Ion (*m/z*)	Product ions (*m/z*)
DHB@MNP-assisted MALDI MS	$[M+Na]^+$	$^{0,2}A_3$, $^{2,4}A_3$, B_2, Y_2, $Y_2/^{0,2}A_3$, Y_1, Y_2/B_2, B_1
MALDI-PSD MS/MS	$[M+Na]^+$	Y_2, $Y_2/^{0,2}A_3$, B_2, $^{0,2}A_3$, Y_1
MALDI-CID MS/MS	$[M+Na]^+$	Y_2, $Y_2/^{0,2}A_3$, B_2, $^{0,2}A_3$
ESI Iontrap MS/MS	$[M+Na]^+$	Y_2, $Y_2/^{0,2}A_3$, B_2
ESI Q-TOF MS/MS	$[M+Na]^+$	Y_2, $Y_2/^{0,2}A_3$

*DHB@MNP-assisted MALDI-MS spectra were obtained at 10000 μg mL^{-1} DHB@MNP as matrix. The unique fragments were highlighted in blue.

4. Optimized structures of 2-FDL

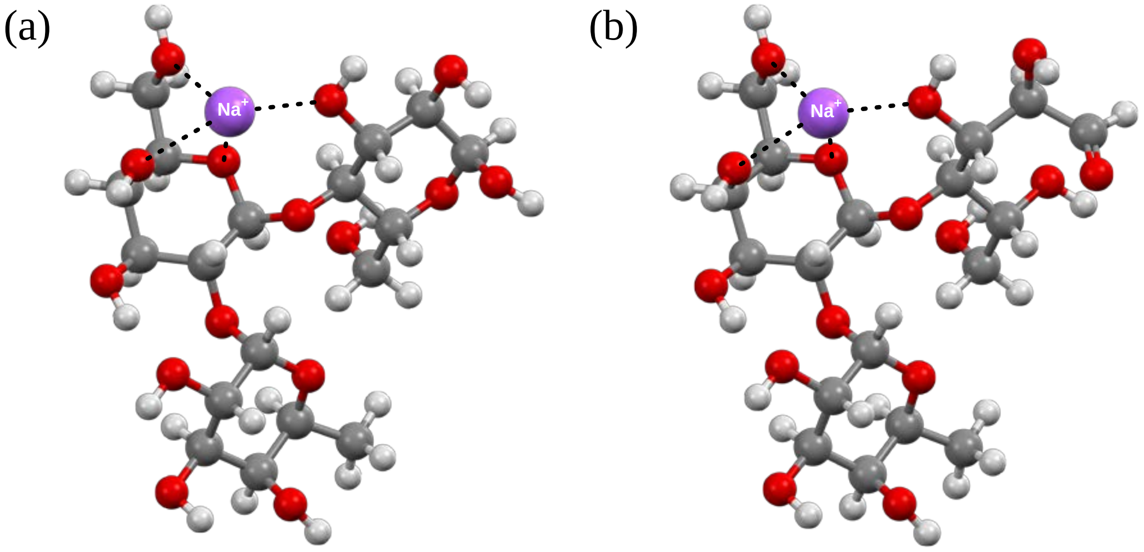

Figure S2. (a) The fully optimized structure of cyclic 2’-fucosyl-D-lactose[Na^+] (c-2FDL [Na^+]), and (b) the fully optimized structure of noncyclic 2’-fucosyl-D-lactose[Na^+] (nc-2FDL [Na^+]).

5. Fragmentation pathways for 2-fucosyl-D-lactose.

For Y_2-type galactose cleavage, a slightly acidic C_2 hydroxyl hydrogen is transferred during fucose cleavage (Scheme S1a); whereas a much less acidic C_2 hydrogen is transferred during galactose B_2-type cleavage (Scheme S1b). The lower activation barrier for fucose cleavage could be due to the stronger acidity of the transferred hydrogen atom in Y_2-type cleavage. In the $^{0.2}A_3$ cross-ring cleavage process, however, the proton of C_3 hydroxyl group in the reducing end was transferred to the nearby aldehyde, forming a six-membered ring transition state (Scheme S1c). Further cleavage from $^{0,2}A_3$ with additional activation energy barrier (nc-$^{2,4}A_3$-TS, 38.22 kcal/mol) will generate $^{2,4}A_3$ (Scheme S1d), which was uniquely formed by our approach, and glycolaldehyde involved migration of hydroxylic proton to aldehyde (Scheme S1c) and the activation energy was 38.22 kcal/mol (nc-$^{2,4}A_3$-TS).

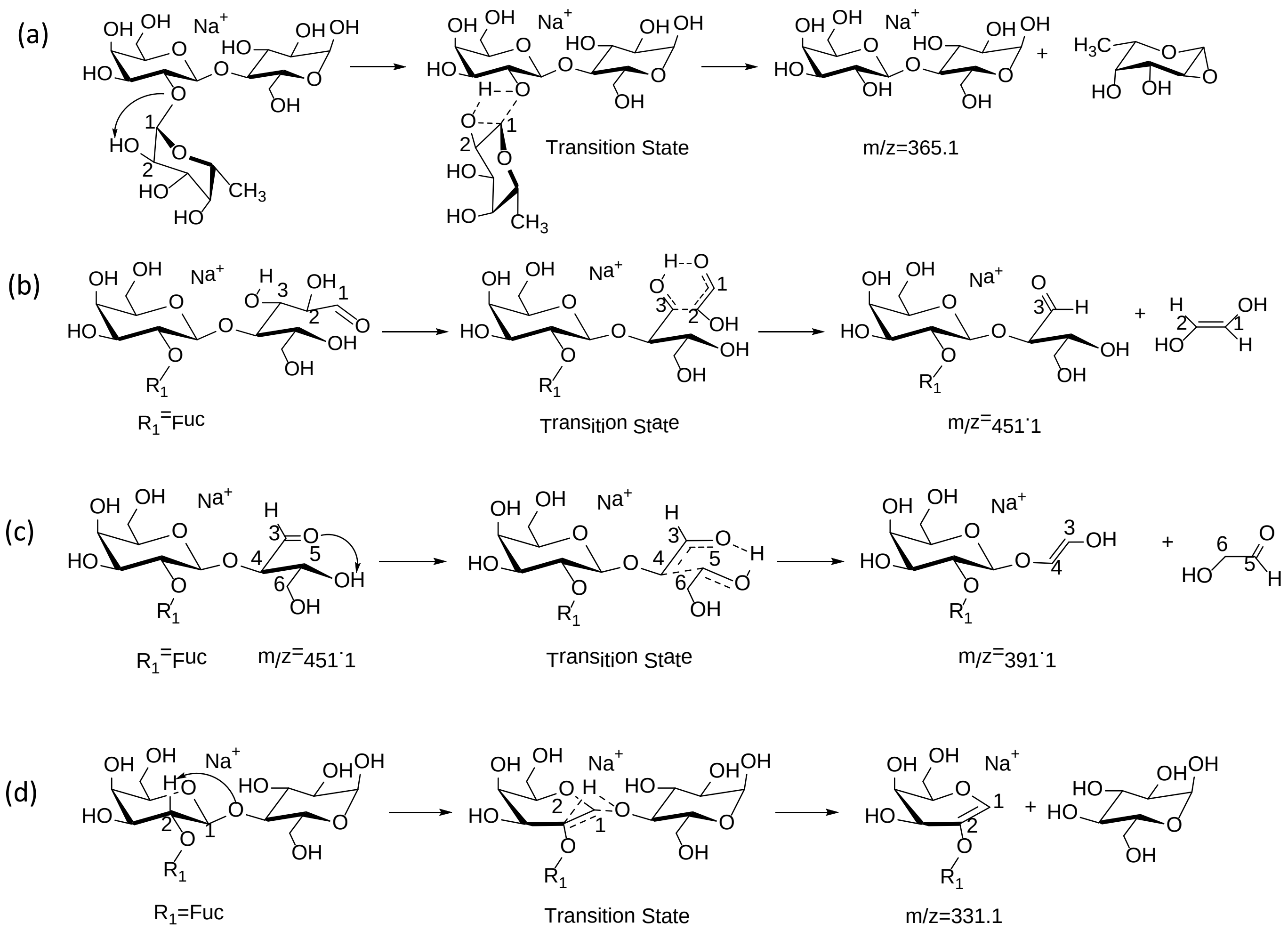

Scheme S1. Fragmentation pathways for (a) Y_2-type, (b) B_2-type, (c) $^{0.2}A_3$-type, (d) $^{2.4}A_3$-type.

6. The mass spectra obtained by multilayer functional nanoparticles.

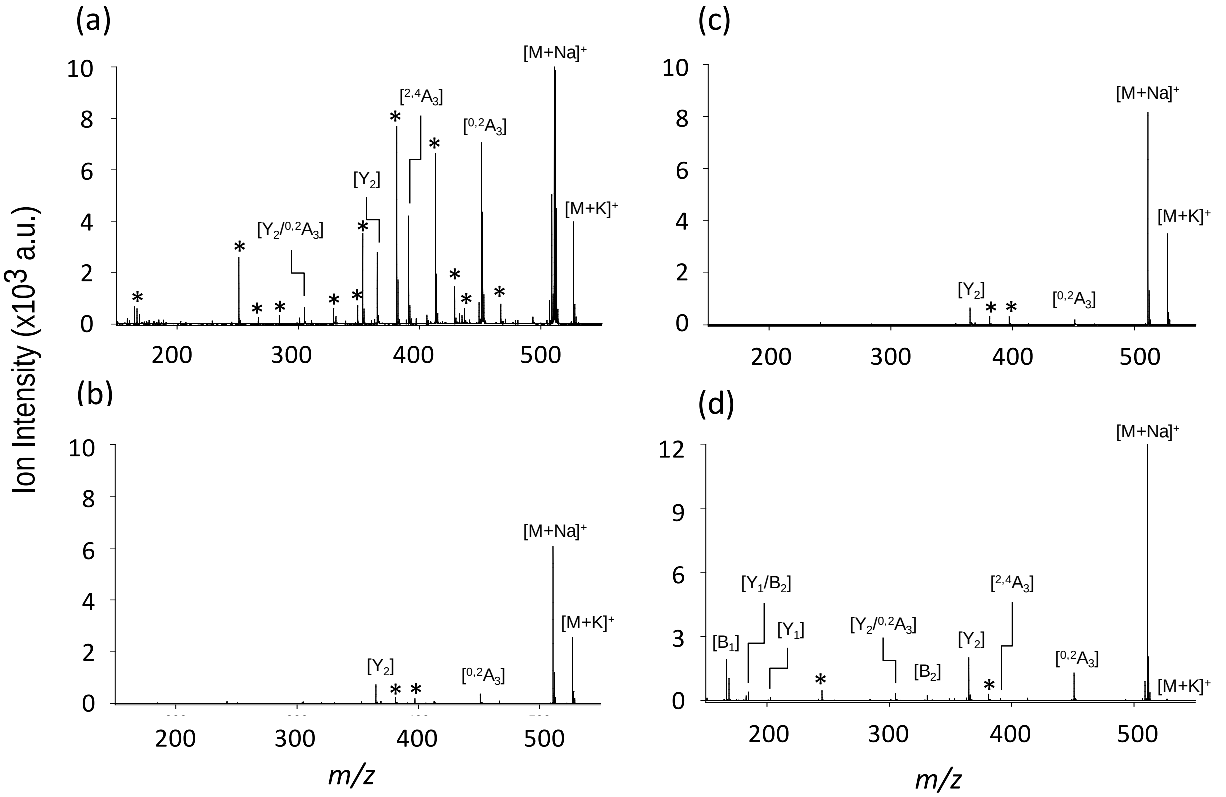

Figure S3. Nanoparticle-assisted MALDI mass spectra of 2FDL in at 300 μg mL^{-1}: (a) Core Fe_3O_4 MNP, (b) SiO_2@MNP, (c) DHB@MNP**.** (d) MALDI MS spectrum at 10000 μg mL^{-1} DHB@MNP. Note the increase in the number of product ions in (d). Laser intensity: 5500. (*) denotes background peaks.

7. Effect of silane thickness on the DHB@MNPs

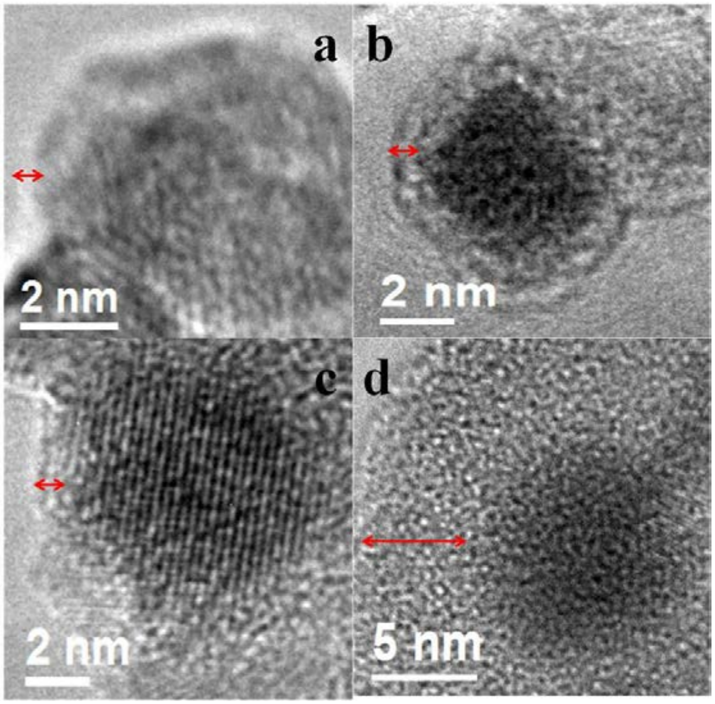

Figure S4. High resolution TEM images of the SiO_2@MNP to study the effect of different silane thickness on the DHB@MNPs . The silane thickness was varied by changing the MNP:TEOS ratio (1:1, 1:2, 1:3.5, and 1:6) of DHB@MNP. The estimated silica thickness is as follows: (a) 0.285 nm–0.54 nm (Ratio: 1). (b) 0.895 nm–1.238 nm (Ratio: 2); (c) 1.278 nm–1.350 nm (Ratio 3.5) and (d) > 10 nm(Ratio 6). (Fringes shown indicates the core magnetic nanoparticle and those without fringes are the amorphous silica coating). Silane thickness is indicated by the red arrows ↔.

8. Alkali metal ion-dependent stability of precursor and fragment ions.

Alkali metal ions, such as Na^+ and K^+, play important role in stabilizing both precursor and fragment ions of oligosaccharides in the MALDI MS[S2-S3]. As such, we attempted to reduce both the free and bound Na^+ ions in the solution and DHB@MNP surface, respectively, by extracting it with 15-crown-5 ether. As shown in Figure S5, we observed that a reduction in precursor ion intensity is accompanied by a decrease in Na^+ ion intensity after extraction by 15-crown-5. After extraction with the Na^+-specific 15-crown-5 ether, we also expected that all Na^+ ions should have been extracted out from the solutions and that this would completely destabilize the precursor ions. On contrary, the precursor ion (**Figure S5b**) and Na^+ ion in the spectra can still be observed even after several extraction (3x) (**Figure S5d**). This might be due to the strong coordination of Na^+ with either the salicylate in the silane shell or the carboxylate ligand in DHB, corroborating with our proposed mechanism (**Figure 6a**).

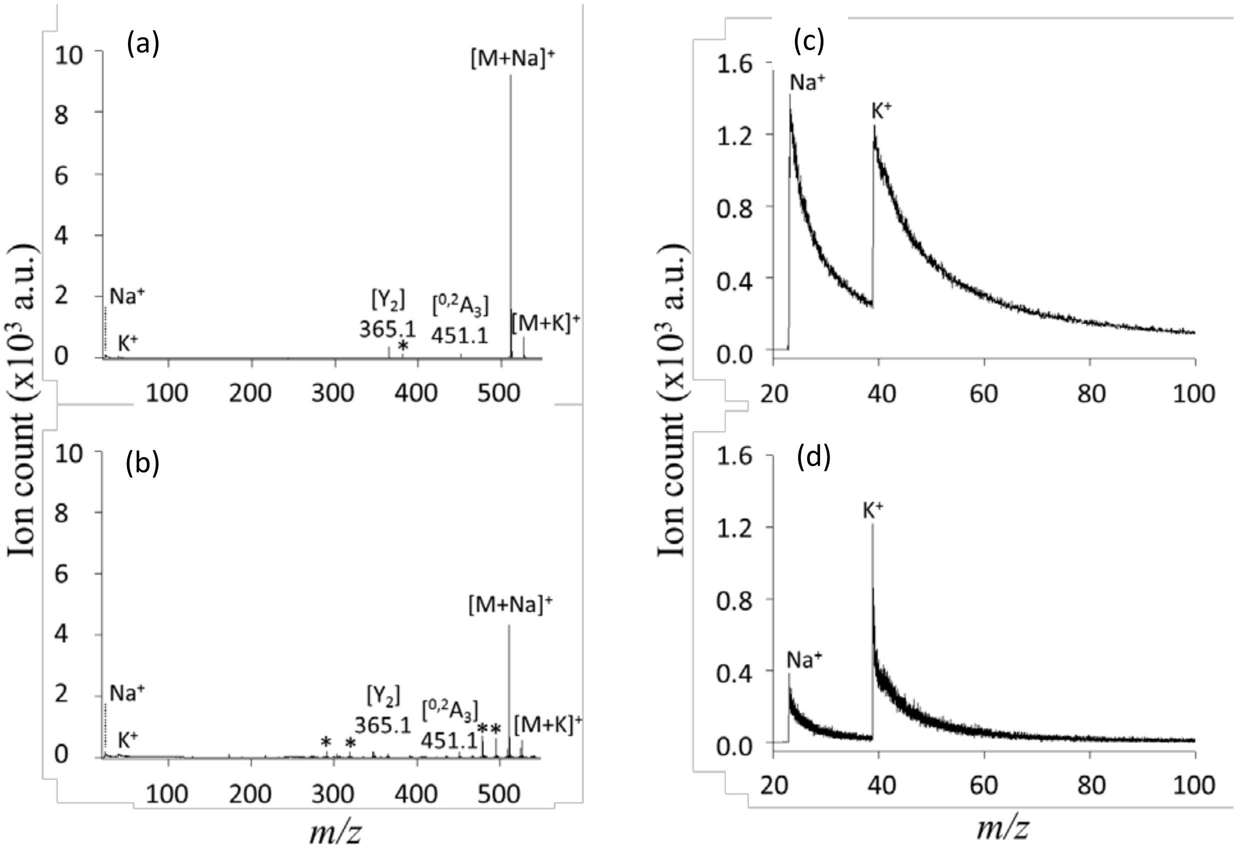

Figure S5. MALDI MS spectra of 2-fucosyl-D-lactose (a) before and (b) after extraction with 15-crown-5. Sodium (Na^+) and potassium (K^+) ions intensities in DHB@MNP solution (c) before and (d) after extraction with 15-crown-5. (*) denotes background peaks. DHB@MNP concentration: 1000 µg mL^{-1}; laser intensity: 3600

9. Differentiation of isomeric oligosaccharides by DHB@MNP-assisted MALDI MS

Table S2. Fragment ions of isomeric oligosaccharides observed by DHB@MNP-assisted MALDI MS

Type of carbohydrate	Parent ion (m/z)	Common Fragment (m/z)	Fingerprint Fragment (m/z)
2-fucosyl-D-Lactose	511.1 ; 527.1	Y_2 (365.1), $Y_2/^{0,2}A_3$ (305.1), Y_1 (203.0), Z_1 (185.0),B_1 (169.0)	$^{0,2}A_3$ (451.1), $^{2,4}A_3$ (391.1), B_2 (331.1),
3-fucosyl-D-Lactose		$Y_{1\alpha}$ (365.1), $Y_{1\alpha}/^{0,2}A_2$ (305.1), $Y_1/Y_{1\alpha}$ (203.1), B_1 (185.0), $B_{1\alpha}$ (169.0)	[$^{0,2}A_2$-C_2H_5O] (405.3), [$Y_{1\alpha}$-H_20] (347.1)
Lewis A trisaccharide	552.1 ; 568.1	$Y_{1\alpha}$ (406.1), $Z_{1\alpha}$ or Y_1 (388.1), C_1 (203.0)	Y_1 (390.1), Z_1 (372.1), $^{3,5}X_1$ (334.1), $Z_1/^{0,4}X_1$ (312.1), $Y_{1\alpha}/Z_1$ (226.1)
Lewis X trisaccharide		$Y_{1\alpha}$ (406.1), $Z_{1\alpha}$ (388.1), C_1 (203.0)	$Y_{1\alpha}/^{0,4}X_1$ (344.1), $Z_{1\alpha}/^{0,4}X_1$ (328.1), $Y_{1\alpha}/^{0,2}A_2$ (305.1), $Y_{1\alpha}/^{2,4}A_2$ (245.1), [$Y_{1\alpha}/Y_1$-H_2O] (208.0), $C_{1\alpha}$ (187.0)
Lewis B tetrasaccharide	698.2 ; 714.4	$Y_{1\alpha}$ or $Y_{2\alpha}$ (552.1), $Z_{2\alpha}$ or $Z_{1\alpha}$ (534.1), $Y_{2\alpha}/Y_{1\alpha}$ (406.1), $Y_{1\alpha}/Z_{2\alpha}$ (388.1), C_1 (349.1)	$^{1,3}X_1$ (490.1), $Z_{1\alpha}/^{0,4}X_1$ (474.1), $Y_1/^{0,4}X_1$ (328.1), C_2 (187.0)
Lewis Y tetrasaccharide		$Y_{1\alpha}$ or $Y_{2\alpha}$ (552.1), Z_2 or $Z_{1\alpha}$ (534.1), $Y_{2\alpha}/Y_{1\alpha}$ (406.1), $Y_{2\alpha}/Z_1$ (388.1), C_1 (349.1)	[$^{2,4}A_2$-H_2O](518.1), Z_1 (372.1), $Z_{1\alpha}/Y_1$ (226.0)

At 10000 μg mL^{-1} DHB@MNP, extensive fragmentation of trisaccharide 3FDL, an isomer of 2FDL, was induced. Several glycosidic and cross-ring dissociations that were characteristic of each isomeric structure and differentiate 2FDL ($^{2,4}A_3$, m/z = 391.1 and B_2 m/z = 331.1) from 3FDL ([$Y_{1\alpha}$-H_2O], m/z = 347.1, and [$^{0,2}A_2$-C_2H_5O], m/z = 405.3) were observed (Figure S6).

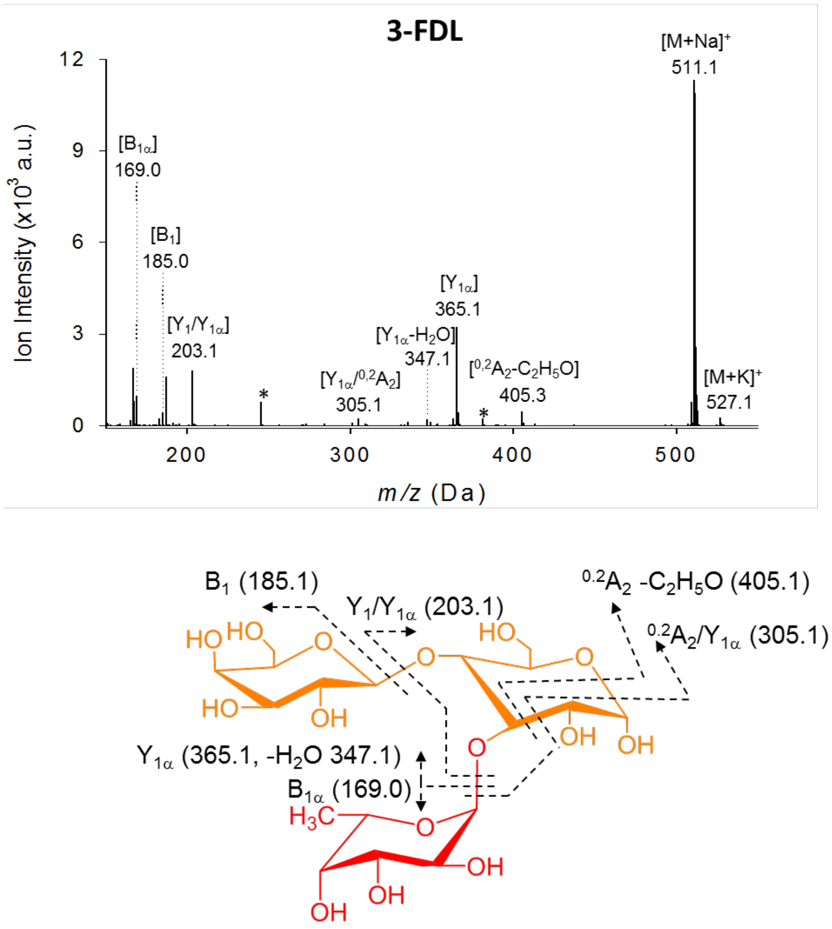

Figure S6. MALDI MS spectra of 3-FDL by using DHB@MNP.

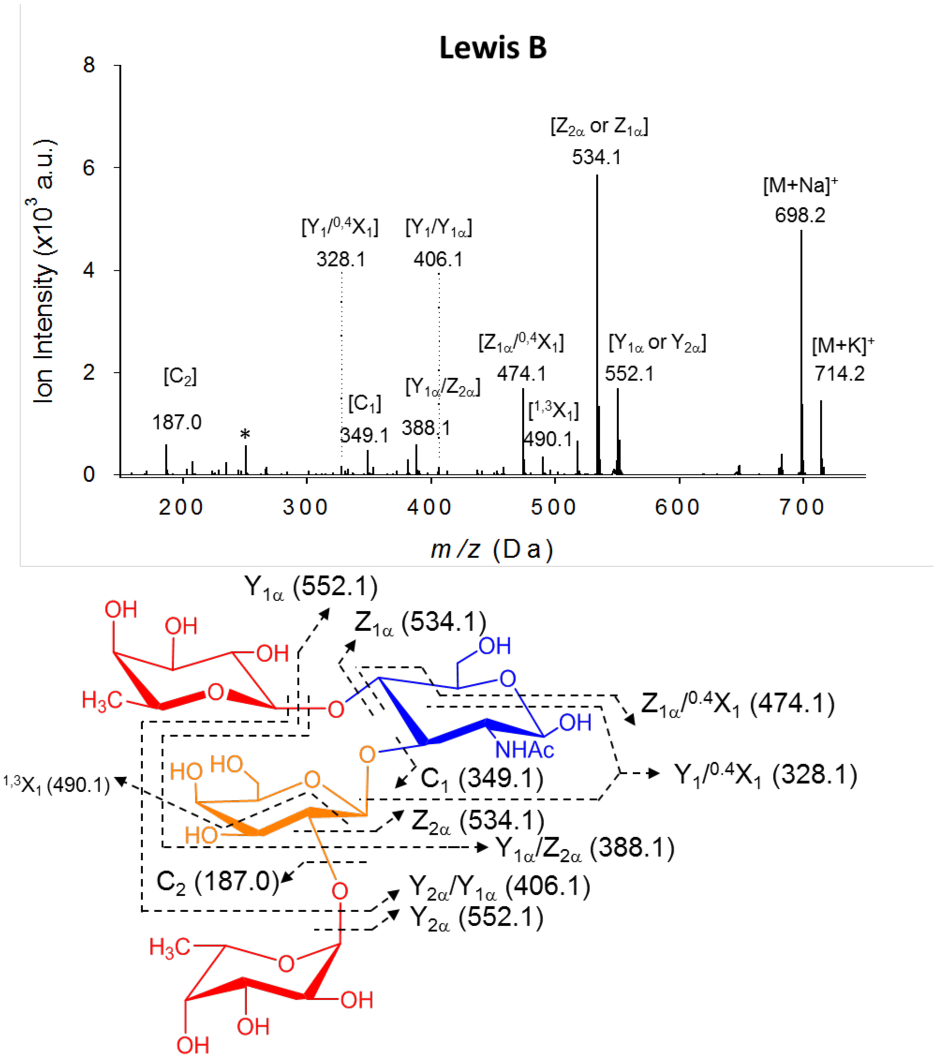

Figure S7. MALDI MS spectra of Lewis B by using DHB@MNP.

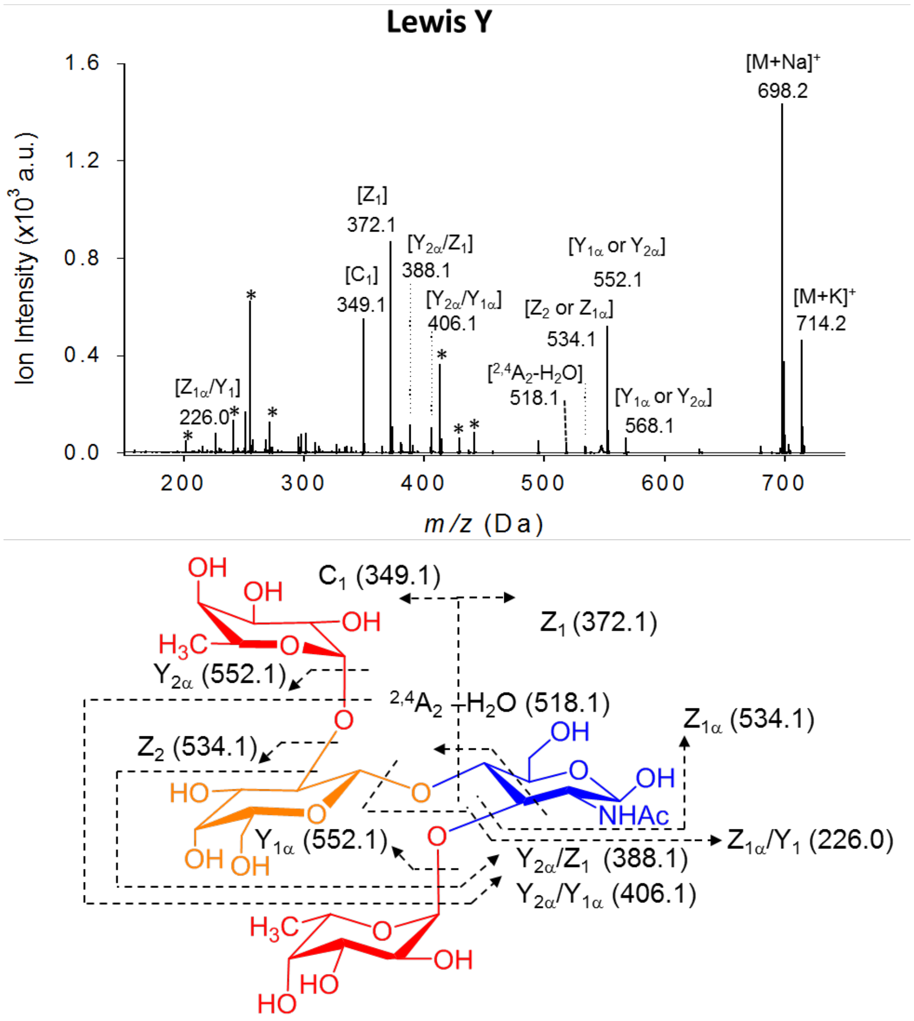

Figure S8. MALDI MS spectra of Lewis Y by using DHB@MNP.

Table S3. Mixture analysis of selected oligosaccharides by the one-step tunable nanomatrix-assisted MALDI MS.

Analyte	Parent ion	Fragment Ion (m/z)	
M1 (Lactose)	365.1 (Na) 381.0 (K)	B_1 (185.0), C_1 (203.0), $^{2,4}A_2$ (245.0), $^{0,2}A_2$ (305.0)	
M2 (3-fucosyl-D-Lactose)	511.1 (Na) 527.1 (K)	$B_{1\alpha}$ (169.0), B_1 (185.0), $Y_1/Y_{1\alpha}$ (203.1), $Y_{1\alpha}/^{0,2}A_2$ (305.1), $[Y_{1\alpha}-H_2O]$ (347.1), $Y_{1\alpha}$ (365.1)	
**Analyte**	**Parent ion**	**Common Fragment**	**Fingerprint Fragment**
M3 (Lewis A)	552.1 (Na) 568.1 (K)	C_1 (203.0), $Z_{1\alpha}$ or Y_1 (388.1), $Y_{1\alpha}$ (406.1)	$Y_{1\alpha}/Z_1$ (226.1), $Z_1/^{0,4}X_1$ (312.1), Z_1 (372.1) Y_1 (390.1)
M5 (Lewis X)		C_1 (203.0), $Z_{1\alpha}$ (388.1), $Y_{1\alpha}$ (406.1)	$C_{1\alpha}$ (187.0), $[Y_{1\alpha}/Y_1-H_2O]$ (208.0), $Y_{1\alpha}/^{2,4}A_2$ (245.1), $Y_{1\alpha}/^{0,2}A_2$ (305.1), $Z_{1\alpha}/^{0,4}X_1$ (328.1), $[^{0,2}X_1 - C_2H_2O]$ (404.1)

10. References

[S1] Domon, B., Costello, C. E. *Glycoconjugate J*. **5**, 397-409, (1988)

[S2] Zhang, J., Ha, T.K., Knochenmuss, R. & Zenobi, R. Theoretical calculation of gas-phase sodium binding energies of common MALDI matrices. *J. Phys. Chem*. **106**, 6610-6617 (2002).

[S3] Zhang, J., Dyachkova, E., Ha, T.K., Knochenmuss, R. & Zenobi, R. Gas-phase Potassium binding energies of MALDI matrices: an experimental and theoretical study. *J. Phys. Chem A.* **107**, 6891-6900 (2003).
